# AGE-FRIENDLY CARE: A FRAMEWORK TO ADDRESS HEALTH DISPARITIES IN NURSING HOMES

**DOI:** 10.1093/geroni/igac059.057

**Published:** 2022-12-20

**Authors:** Leland Waters, Diane Berish, Annie Rhodes, Michele Bellantoni

**Affiliations:** Virginia Commonwealth University, Richmond, Virginia, United States; Pennsylvania State University, University Park, Pennsylvania, United States; Virginia Commonwealth University, Richmond, Virginia, United States; Johns Hopkins University, Baltimore, Maryland, United States

## Abstract

Three Geriatrics Workforce Enhancement Programs (Johns Hopkins, Penn State and Virginia Commonwealth) within Health and Human Services Region-3, implemented a project providing tailored educational content designed to meet the regional needs of nursing home staff, residents, and carepartners within the context of the COVID-19 pandemic. Semi-Structured, recorded focus groups were conducted with direct care workers, and family and resident counsels. All groups were interviewed about familiarity and comfort with the 3Ds (Dementia, Delirium, and Depression) as well as what mattered most to them as workers, residents and care partners. The overall goal was to enhance development of the nursing home workforce thus creating a meaningful clinical improvement in care. We aim to go beyond the improvement of routine clinical care and address the long-standing behavioral and mental health disparities observed in this resident population. Policy implications around education, engagement of workforce, resident, and carepartner voices will be discussed.

